# Simulation and Experimental Validation of Long-Wave Infrared Polarization Characteristics Based on the Smith-pBRDF Model

**DOI:** 10.3390/s26206485

**Published:** 2026-10-13

**Authors:** Shengbo Liu, Yingchun Li, Haijing Zheng, Laixian Zhang, Rong Li, Huichao Guo, Yang Zhao, Mengci Li, Zhen Deng, Rui Zhu

**Affiliations:** 1 Space Engineering University, No. 1 Bayi Road, Huairou District, Beijing 101416, China; 15941724315@163.com (S.L.); zhanglaixian@pku.edu.cn (L.Z.);; 2 National Key Laboratory of Space Target Awareness, No. 1 Bayi Road, Huairou District, Beijing 101416, China

**Keywords:** long-wave infrared polarization, polarized bidirectional reflectance distribution function (pBRDF), Smith masking–shadowing function, thermo-optic effect, microfacet model

## Abstract

**Highlights:**

**What are the main findings?**

- A Smith masking–shadowing function is embedded into a polarized BRDF framework, eliminating the grazing-angle divergence of conventional models in long-wave infrared polarization simulation.
- For silicon carbide and alumina measured at three temperatures, the proposed three-parameter Smith model matches division-of-focal-plane measurements with the lowest errors. The original P–G model—which obtains its emission polarization from the smooth-interface Fresnel relation, without the microfacet distribution—overestimates the DoLP by roughly an order of magnitude, whereas the five-parameter Minnaert model is accurate but requires more parameters. The Smith model attains the lowest AICc and BIC for both materials, demonstrating improved physical fidelity together with parameter efficiency.

**What are the implications of the main findings?**

- The model provides physically consistent long-wave infrared polarization signatures that support infrared target recognition and camouflage discrimination.
- The validated forward-simulation framework can guide the design and performance assessment of infrared polarization sensing systems.

**Abstract:**

Long-wave infrared (LWIR, 8–14 μm) polarization detection supports target recognition, but measuring target polarization characteristics is costly and sample-limited, and existing polarized bidirectional reflectance distribution function (pBRDF) models suffer from energy divergence at grazing angles, over-reliance on empirical parameters, and neglect of the temperature dependence of the complex refractive index. To address these issues, a Smith-pBRDF analytical model is proposed: a closed-form Smith masking–shadowing function embedded in the microfacet specular term suppresses grazing-angle divergence; two-dimensional directional–hemispherical reflectance (DHR) integration coupled with Kirchhoff’s law models rough-surface emission polarization; and wavelength-dependent optical constants are integrated over the 8–14 μm detector band, with the thermo-optic effect included through dynamic complex-refractive-index evolution. Division-of-focal-plane (DoFP) LWIR polarization imaging of silicon carbide and alumina at 338 K, 358 K and 378 K over viewing angles of 0°–80° is used to compare the proposed model with the original Priest–Germer (P–G) and the Minnaert-modified models. The measured degree of linear polarization (DoLP) increases with both viewing angle and temperature; this rise is governed primarily by the Planckian target–background radiance contrast, while the thermo-optic term provides a secondary refinement. The original P–G model—which obtains its emission polarization from the smooth-interface Fresnel relation, neglecting the microfacet distribution—overestimates the DoLP by roughly an order of magnitude (RMSE 0.036–0.089). The Minnaert and Smith models both reproduce the measurements well, with RMSE 0.0004–0.0011 for alumina and 0.0032–0.0070 for silicon carbide. The proposed three-parameter Smith model attains the lowest AICc and BIC for both materials while using fewer parameters than the Minnaert model, demonstrating improved physical fidelity and parameter efficiency.

## 1. Introduction

Infrared polarization detection, by virtue of its distinctive ability to capture polarization information, can accurately discriminate the differences in polarization characteristics between targets and backgrounds, effectively compensating for the weaknesses of conventional infrared intensity detection, namely low target discriminability and weak anti-interference capability in complex environments. It has consequently become a research focus in fields such as military target recognition, camouflage and anti-camouflage, photoelectric detection, and remote sensing [1,2].

Real measurement environments are complex and variable, and constrained by the high cost of high-precision polarization cameras and by experimental conditions; measured data samples remain scarce and can hardly meet the research demands under complex operating conditions. Therefore, developing high-precision theoretical modeling and simulation of infrared polarization characteristics has become an effective approach to addressing these problems. By constructing analytical models, the physical limitations of experimental hardware and environments can be overcome to enable theoretical prediction of target polarization characteristics under diverse operating conditions, providing a reference for the design of infrared polarization detection systems and the optimization of target recognition algorithms [3,4,5,6].

The polarized bidirectional reflectance distribution function (pBRDF) is the fundamental mathematical model describing the infrared polarized radiation characteristics of target surfaces, and it plays a key role in infrared polarization detection simulation and target characteristic analysis; its modeling accuracy substantially affects the engineering performance of detection technology [7]. Pursuing the optimization and innovation of pBRDF models through high-precision theoretical modeling is therefore of important theoretical value and engineering significance for advancing infrared polarization detection.

Torrance and Sparrow decomposed the reflection of a target surface into specular and diffuse reflection and proposed the classical T–S model [8]. Flynn incorporated the Mueller and Jones matrices into the radiative transfer equation, extending the conventional non-polarized BRDF model to the polarization domain [9]. Priest and Germer combined the T–S model with the Mueller matrix and proposed the polarized bidirectional reflectance distribution function model (the P–G model) [10], establishing a representative foundational model in this field. Since then, numerous researchers have continued to refine the model and extend its scenarios. In terms of fundamental analytical expressions, Chen et al. derived a mathematical model of target infrared polarization characteristics based on the T–S model [11], while Liu et al. employed the Mueller matrix to improve the analytical description of the degree of polarization of infrared radiation from rough surfaces [7]. Addressing the geometric attenuation and masking effects of microfacets, Hyde et al. introduced geometric attenuation and volume-scattering characteristics, improving the performance of the P–G model at large reflection angles [12]; Liu et al. and Zhu et al. proposed, respectively, an integral masking-function model and a corrected spontaneous-emission polarization model for metal surfaces [13,14]; Wang et al. adopted a three-component assumption for the Blinn masking effect to improve model adaptability [15]; and Wang explored approaches to improving modeling at large incidence angles by introducing the Cauchy probability distribution function [16]. Furthermore, for multi-component coupling and complex detection conditions, Yang et al. combined Kubelka–Munk theory to propose a three-component pBRDF model, improving the fit to specific measured data [5]; Yang et al. analyzed the influencing factors of target infrared polarization characteristics on the basis of the thermal radiation law and performed experimental verification [17]; Yan et al. derived a mixed infrared linear-polarization-degree model for high-temperature rough surfaces, providing a reference for polarization modeling in extreme-temperature scenarios [18]; and Ming et al. proposed an enhanced p-BRDF model based on Minnaert scattering and explored a modeling approach for target polarization characteristics in sea-fog environments [19].

Despite this series of achievements, in real detection environments, the infrared polarized radiation from target surfaces is affected by multiple factors, including the material thermodynamic state, microscopic geometric morphology, and observation geometry, and conventional pBRDF models still exhibit several limitations in practical application. First, the classical P–G model is prone to energy divergence in large-angle grazing scenarios, leading to deviations in polarization simulation results. Second, some improved models suppress divergence by introducing empirical fitting parameters; although this enhances fitting accuracy to a certain extent, the physical meaning of such parameters is insufficiently clear, and overfitting may arise for unknown materials. Third, most models treat the material complex refractive index as a static constant, neglecting the temperature dependence induced by the thermo-optic effect, which may produce simulation errors when the target undergoes substantial temperature variation.

To address these limitations, this paper proposes an analytical Smith-pBRDF model. The GGX distribution and the Smith masking–shadowing function are established microfacet results and are not presented as new; the contribution is their integration into a long-wave infrared thermal-emission polarization framework, which differs from existing passive, room-temperature, reflection-only microfacet models in three concrete ways: (1) the microfacet reflection is closed through the Kirchhoff relation and the DHR to model the polarized spontaneous emission of a rough surface; (2) the complex refractive index is a dynamic, temperature-dependent quantity, and the Stokes components are integrated over the 8–14 μm band; and (3) the closed-form Smith factor is used with a strictly consistent GGX distribution under the energy-conservation constraint (ks + kd ≤ 1), reaching competitive accuracy with only three fitted parameters. The model thereby reduces reliance on empirical parameters and provides theoretical support for long-wave infrared polarization detection in complex environments.

## 2. Theoretical Model of Infrared Polarization Characteristics

The infrared polarized radiation of a target generally results from the superposition of two parts: the surface reflection of environmental background radiation and the target’s own thermal emission. To accurately describe the polarization characteristics of a target under a given observation geometry, establishing a theoretical computational model with high fidelity is a critical step. This section proceeds from the two parts of reflection and emission to derive the infrared polarized radiation laws of target surfaces step by step. First, to address the divergence limitation of the classical pBRDF at large grazing angles, an analytical geometric masking function is introduced to construct a corrected reflection polarization model. Then, on the basis of energy conservation and the thermal radiation law, the emission polarization characteristics of rough surfaces are derived and reconstructed, thereby forming a complete theoretical model of infrared polarization.

### 2.1. Reflection Component

#### 2.1.1. Classical pBRDF

The classical theory of the pBRDF is built on the microfacet assumption, which postulates that a macroscopically rough surface is composed of innumerable microfacets that are locally smooth. Early studies combined the Mueller matrix with the bidirectional reflectance distribution function theory, laying the foundation for quantifying polarized reflection behavior. On this basis, Priest and Germer proposed the classical P–G model, pointing out that the macroscopic polarized reflection of a rough surface is dominated primarily by the specular reflection process of local microfacets [10].

In the classical P–G model, the specular reflection polarization component is computed as:

(1) $$f_{spec,j}=\frac{D\left(\alpha \right)\cdot R_{j}\left(\theta_{local},m\right)}{4\cos\theta_{i}\cos\theta_{r}}$$

where $f_{spec,j}$ is the specular reflection component of the target surface; the subscript j is the polarization-state identifier, which takes s and p to denote the polarization components perpendicular and parallel to the plane of incidence, respectively; $D\left(\alpha \right)$ is the microfacet normal distribution function describing the morphology of the random rough surface, assumed to follow the Gaussian (Beckmann) distribution in the P–G baseline; α is the angle between the normal of a local microfacet and the mean normal of the macroscopic surface; $R_{j}$ is the Fresnel reflectance of the local microfacet; $\theta_{local}$ is the actual incidence angle on the local microfacet; m is the complex refractive index of the material; and $\theta_{i}$ and $\theta_{r}$ are the incidence zenith angle and the observed reflection zenith angle in the macroscopic coordinate system.

The classical P–G model offers good theoretical reference value at small and moderate observation angles. However, as can be seen from Equation (1), the denominator contains the cosine term of the macroscopic reflection zenith angle. When the line of sight gradually approaches a large grazing angle, this cosine term tends to zero, which readily drives the theoretically computed reflected energy toward infinity; such energy divergence deviates to some extent from real physical laws.

#### 2.1.2. Smith-pBRDF Model

To remedy the theoretical deficiency of the classical pBRDF model at large observation angles, this paper reconstructs the reflection model by introducing a geometric masking function. On a real rough physical surface, as the incidence or observation angle increases, adjacent microfacets occlude one another, preventing part of the light from reaching the observer. The Smith geometric masking function, combined here with the long-tailed GGX (Trowbridge–Reitz) slope distribution, can effectively quantify this mutual occlusion caused by microscopic undulations.

The bidirectional Smith masking–shadowing function, which accounts for the correlated occlusion along the incident and reflected paths, is expressed as:

(2) $$G_{smith}\left(\theta_{i},\theta_{r},\sigma \right)=\frac{1}{1+\Lambda \left(\theta_{i},\sigma \right)+\Lambda \left(\theta_{r},\sigma \right)}$$

where $G_{smith}$ is the computed bidirectional geometric masking probability; σ is a dimensionless GGX roughness scale parameter (not a measured RMS slope); and Λ is a unidirectional correction factor related to both the light propagation direction and the roughness scale.

The mathematical expression of this unidirectional correction factor is:

(3) $$\Lambda \left(\theta ,\sigma \right)=\frac{1}{2}\left[\sqrt{1+\sigma^{2}\tan^{2}\theta }-1\right]$$

where the variable θ is a generic zenith-angle placeholder that can be replaced by either the incidence or the reflection zenith angle during computation.

The physical essence of the unidirectional correction factor Λ in Equation (3) is the area-integrated expectation of the occlusion probability of microscopic inclined facets. The theoretical framework of this analytical expression was first proposed by Smith [20]; the square-root algebraic form adopted in this paper is a rigorous analytical solution of the Smith probability integral equation obtained under the assumption that the microfacet slopes follow the long-tailed Trowbridge–Reitz (GGX) probability density distribution [21]. Compared with the numerical solution involving complicated error functions under the conventional Gaussian assumption, this analytical expression strictly preserves the self-consistency of the statistical probability while effectively reducing the computational complexity.

To keep the statistical description fully self-consistent with the analytic Smith factor, the microfacet normal distribution of the proposed model is the Trowbridge–Reitz (GGX) distribution [21], defined as:

(4) $$D_{G}GX\left(\alpha ,\sigma \right)=\sigma^{2}/\left(\pi cos^{4}\alpha {\left(\sigma^{2}+tan^{2}\alpha \right)}^{2}\right)$$

where σ is a dimensionless roughness scale parameter, and α is the tilt angle of a local microfacet. This GGX distribution differs from the Gaussian NDF retained in the P–G and Minnaert baselines, and it is precisely the distribution under which the closed-form Smith factor in Equations (2) and (3) is derived.

Once the masking probability is obtained, it is embedded into the reflection polarization matrix, and the corrected specular reflection polarization component is updated as:

(5) $$f_{spec,j}=\frac{D\left(\alpha ,\sigma \right)\cdot R_{j}\left(\theta_{local},m\right)\cdot G_{smith}\left(\theta_{i},\theta_{r},\sigma \right)}{4\cos\theta_{i}\cos\theta_{r}}$$

After introducing this masking correction term, under large-angle grazing conditions, the masking probability in the numerator converges to zero at a faster rate.

In addition, the complex refractive index of a material generally exhibits temperature dependence in engineering practice [22]. To reflect the modulation of photo-thermal coupling by temperature variation, this paper formulates it as a dynamic variable related to the current physical temperature of the target:

(6) $$m\left(T_{obj}\right)=\left(n\left(T_{ref}\right)+\frac{dn}{dT}\Delta T\right)+i\cdot \left(k\left(T_{ref}\right)+\frac{dk}{dT}\Delta T\right)$$

where $T_{obj}$ is the current physical temperature of the target; $T_{ref}$ is the experimentally calibrated reference temperature; n and k are the real part of the refractive index and the imaginary part of the extinction coefficient at the reference temperature, respectively; dn/dT is the thermo-optic coefficient; dk/dT is the extinction temperature coefficient; and ΔT is the difference between the current temperature and the reference temperature.

Having defined the dynamic complex refractive index and combined the various corrections, both specular and diffuse reflection must be considered. The complete Smith-pBRDF model constructed in this paper is defined as:

(7) $$f_{r,j}\left(\theta_{i},\phi_{i},\theta_{r},\phi_{r}\right)=k_{s}\cdot f_{spec,j}+\frac{k_{d}}{\pi }$$

where $f_{r,j}$ is the reflected polarization component; $\phi_{i}$ and $\phi_{r}$ are the macroscopic incidence and reflection azimuth angles, respectively; and $k_{s}$ and $k_{d}$ are the specular and diffuse reflection weight coefficients. The actual directional–hemispherical reflectance is obtained by integrating the polarized reflection function over the hemisphere, ρ_j = k_s ρ_spec,j + k_d, rather than being equal to the sum k_s + k_d; the weight coefficients are constrained by energy conservation (k_s + k_d ≤ 1).

### 2.2. Emission Component

#### 2.2.1. Infrared Polarized Radiation Model

In infrared detection, the energy reaching the camera from a target includes not only the reflection of environmental radiation but also the spontaneous emission of the material. According to Kirchhoff’s law of thermal radiation, for a body in thermodynamic equilibrium, the sum of its directional emissivity and the hemispherical total reflectance in that direction is identically equal to one [23].

Incorporating the reflection compensation from the environmental background, the total infrared polarized radiance of the target in a given observation direction can be derived as:

(8) $$L_{tot,j}\left(\theta_{r}\right)=L_{r,j}^{Source}+\epsilon_{j}\left(\theta_{r}\right)\cdot L_{bb}\left(T_{obj}\right)+\left(1-\epsilon_{j}\left(\theta_{r}\right)\right)\cdot L_{bb}\left(T_{bg}\right)$$

where $L_{tot,j}$ is the polarized component of the total radiance of the target in the given observation direction; the subscript j denotes the polarization state, which takes s and p; $\theta_{r}$ is the observed reflection zenith angle; $L_{r,j}^{Source}$ is the reflected radiance from any active light source that may exist in the scene, which is usually set to zero in a passive darkroom environment; $\epsilon_{j}$ is the polarized directional-emissivity component of the rough surface in the corresponding observation direction; $L_{bb}$ is the ideal blackbody radiance computed according to Planck’s blackbody radiation law; $T_{obj}$ is the current physical temperature of the target; and $T_{bg}$ is the effective physical temperature of the environmental background.

After the total radiance in the different polarization states is obtained, the macroscopic degree of linear polarization can be calculated as:

(9) $$DoLP_{tot}=\frac{L_{tot,s}-L_{tot,p}}{L_{tot,s}+L_{tot,p}}$$

where $DoLP_{tot}$ is the macroscopic total degree of linear polarization of the target surface.

#### 2.2.2. Directional–Hemispherical Reflectance (DHR) Integration

In early studies of the thermal-radiation polarization characteristics of targets, modeling relied on the ideal smooth-interface assumption and the Kirchhoff thermal-radiation conservation relation, converting the polarized emissivity of the material indirectly through the Fresnel polarized reflection coefficients. This approach did not incorporate the modulation of the radiation field by rough surfaces; it also neglected the rotation of the polarization reference coordinate system caused by the random tilt of microfacets and the resulting redistribution of polarization states, ultimately producing deviations between the theoretical simulation and the actual measurement of the degree of spontaneous-emission polarization.

To capture the depolarization of rough surfaces induced by the dispersion of microfacet slopes, this paper employs the DHR to rigorously derive the emission polarization characteristics. According to the Helmholtz reciprocity principle, the total reflectance of a target surface in a particular observation direction is numerically equivalent to the integral of its BRDF over the entire hemispherical space when that observation direction is taken as the incidence source.

The two-dimensional hemispherical integration model of the DHR is established as:

(10) $$\rho_{j}^{DHR}\left(\theta_{r},\lambda \right)=\int_{0}^{2\pi }\int_{0}^{\pi /2}f_{r,j}\left(\theta_{r},\theta ,\phi ,\lambda \right)\cos\theta \sin\theta d\theta d\phi$$

where $\rho_{j}^{DHR}$ is the polarized directional–hemispherical-reflectance component; λ is the infrared operating wavelength; $f_{r,j}$ is the complete polarized reflection function derived in the previous subsection; and θ and ϕ within the integration domain are the infinitesimal zenith and azimuth angles in the hemispherical space.

The above integration realizes the physical-mechanism mapping from reflection to emission. Substituting the analytical reflection model constructed above into this integral means that the thermo-optic evolution of the complex refractive index participates in the hemispherical energy accumulation; the dominant temperature dependence of DoLP, however, arises from the Planckian target–background radiance contrast, while the thermo-optic term provides a secondary, high-order refinement. Meanwhile, the embedded Smith geometric masking function effectively constrains the energy divergence among microfacets as the integration approaches the grazing-angle boundary, ensuring the convergence of the entire two-dimensional integration.

Because the integral contains the nonlinear masking correction function and probability-distribution terms and is difficult to solve analytically, a high-density two-dimensional trapezoidal numerical quadrature algorithm is used in the simulation to discretize the hemispherical space into a grid. Once the high-fidelity directional–hemispherical reflectance is obtained, it is substituted into the generalized Kirchhoff law to compute the directional emissivity of the rough surface:

In each hemispherical sampling direction, the local Fresnel reflection is evaluated in the local plane of incidence and then projected onto the global s–p polarization basis of the observation direction. The cross-polarized term that contains the sine of the basis rotation cancels upon azimuthal integration for an isotropic surface, so the projection is implemented at the intensity level; the remaining s–p mixing represents the depolarization caused by the dispersion of microfacet slopes.

(11) $$\epsilon_{j}\left(\theta_{r}\right)=1-\rho_{j}^{DHR}\left(\theta_{r}\right)$$

Substituting the directional emissivity obtained from this equation into the radiance equation established above closes the loop from the microscopic reflection mechanism to the macroscopic emission characteristics. At the theoretical level, this method captures the macroscopic depolarization caused by microfacet slope dispersion and geometric masking (rather than genuine subsurface volume scattering) and provides a computational basis for improving the overall fidelity of the theoretical model of infrared polarization characteristics.

#### 2.2.3. Broadband Spectral Integration

The DoFP camera responds over the full 8–14 μm band, whereas the preceding equations are formulated at a single wavelength. The Stokes components recorded by the detector are therefore spectrally integrated over the band:

(12) $$S_{k}=\int_{\left(8\mu m\right)}^{\left(14\mu m\right)}s_{k}\left(\lambda \right)R\left(\lambda \right)d\lambda$$

where k = 0, 1, 2, λ1 = 8 μm and λ2 = 14 μm, s_k(λ) are the monochromatic Stokes components of the target, and R(λ) is the combined detector–lens spectral responsivity. The band-integrated degree of linear polarization is then:

(13) $$DoLP_{b}and=\surd \left(S_{1}^{2}+S_{2}^{2}\right)/S_{0}$$

The wavelength-dependent optical constants *n*(λ,T) and *k*(λ,T), together with the Planck radiance L_bb(λ,T), are evaluated at each wavelength in this integration. The uncooled VOx microbolometer is approximately spectrally flat over the band. Because the combined detector–lens responsivity was not available, a flat R(λ) is adopted, and the sensitivity to alternative response shapes is quantified: across flat, trapezoidal, and bell-shaped response curves, the RMSE changes by at most 0.0002 for silicon carbide and 0.0001 for alumina (Section 4.2), confirming only a weak dependence of the band-integrated DoLP on the response shape within the tested forms.

Within the spectrally integrated model, the increase in DoLP with target temperature is governed primarily by the Planckian target–background radiance contrast; the thermo-optic evolution of *n*(λ,T) and *k*(λ,T) provides a secondary modulation of the physical DoLP (on the order of 10^−5^–10^−4^, reaching about 1.2 × 10^−4^ at ΔT = 90 K near grazing angles), which is retained because it explicitly represents the assumed temperature dependence of the optical constants rather than acting as the primary driver.

## 3. Experiments

To examine model performance under real conditions, this section describes the hardware composition of the polarization imaging system and the physical-parameter settings of the experimental samples, providing the experimental basis for subsequent analysis.

### 3.1. Experimental System

**Figure 1:**
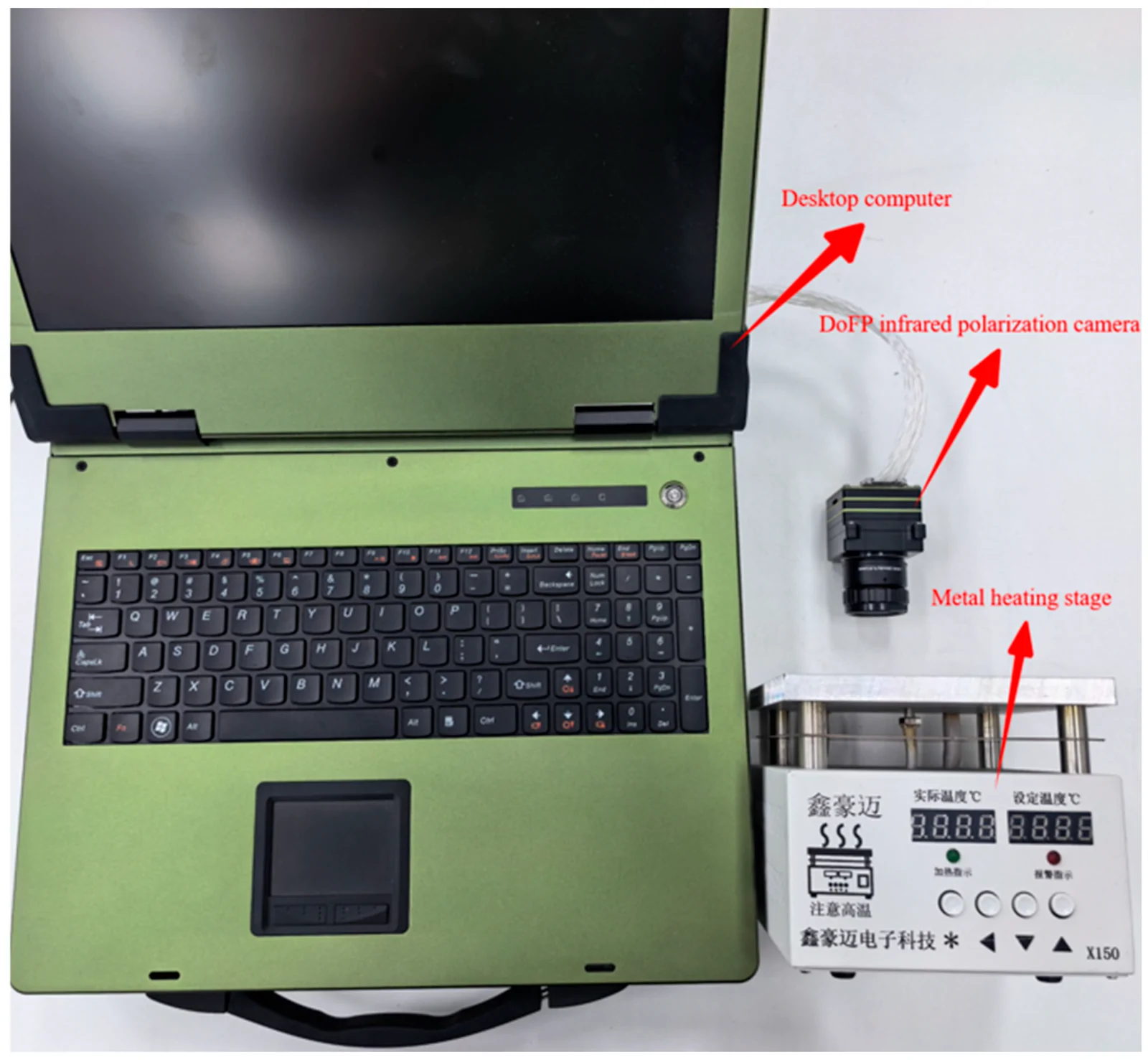
Experimental equipment.

The experimental system consists of three parts: a target heating stage, an infrared polarization camera, and a computer. The experimental sample was placed on a numerically controlled constant-temperature heating stage (Model X150, Shenzhen Xinhaomai Electronic Technology Co., Ltd., Shenzhen, China; temperature-control accuracy of ±1 K). A sensor attached to the stage surface monitors and stabilizes the physical temperature of the sample in real time through a control algorithm. The system equipment is shown in Figure 1.

**Figure 2:**
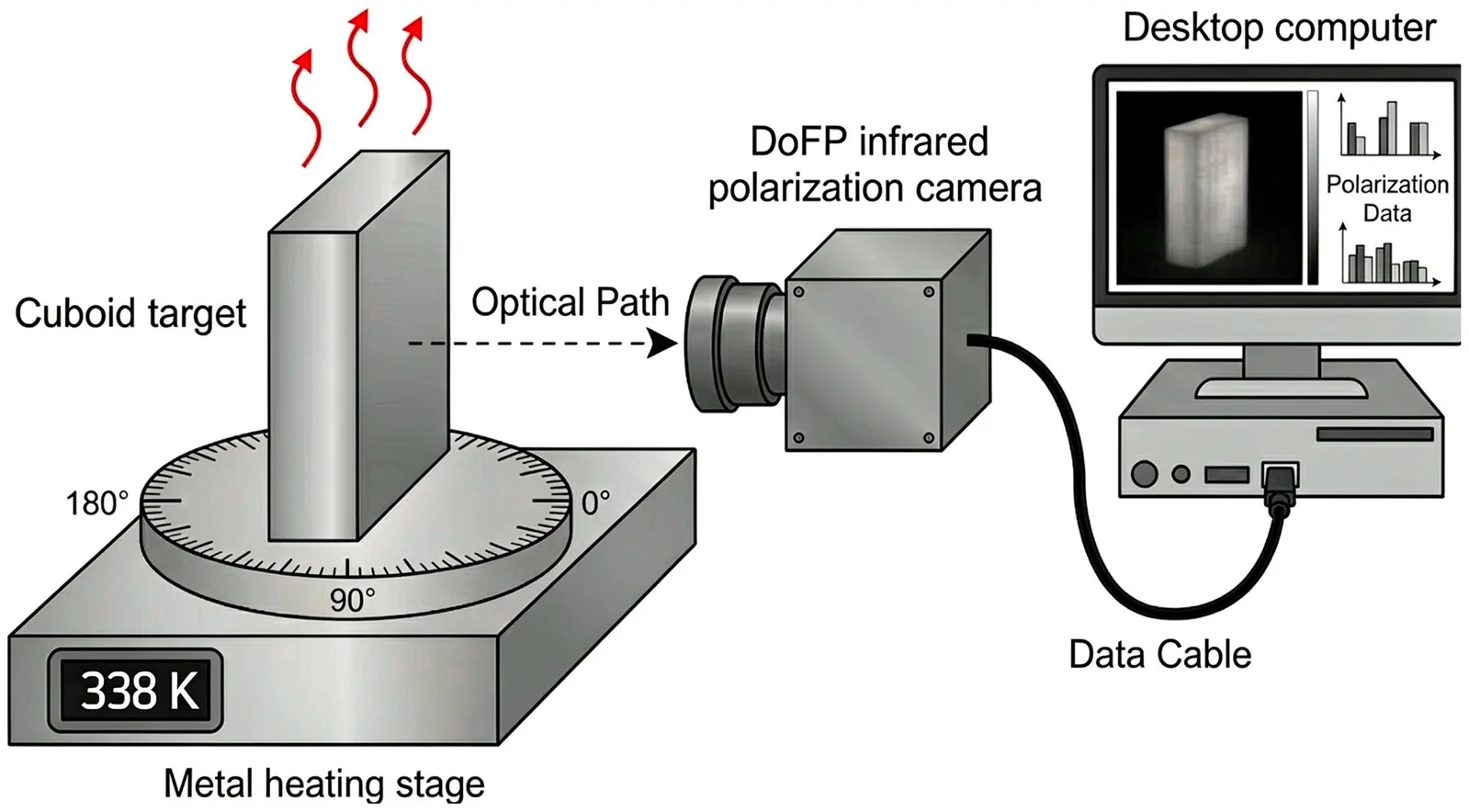
Schematic of the polarization-characteristic experimental system.

During the experiment, the target material was placed vertically on the heating stage (which is engraved with viewing-angle scales). After the operating temperature of the stage was set, a temperature-measurement device was used to verify that the surface temperature of the material reached the preset value, and a division-of-focal-plane infrared polarization camera was used to acquire the target data. A schematic of the experimental system is shown in Figure 2.

A long-wave division-of-focal-plane infrared polarization imaging device was used in this experiment; its detection core is an uncooled vanadium-oxide focal-plane-array detector. Table 1 lists the specifications of the infrared polarization detector.

For each viewing angle, the DoFP camera records four linearly polarized intensity images I_0_, I_45_, I_90_, and I_135_, corresponding to the micro-polarizer axes at 0°, 45°, 90°, and 135°. The Stokes parameters are recovered from these images as:

(14) $$S_{0}=\left(I_{0}+I_{45}+I_{90}+I_{135}\right)/2,S_{1}=I_{0}-I_{90},S_{2}=I_{45}-I_{135}$$

The degree of linear polarization is then computed as DoLP = √(S_1_^2^ + S_2_^2^)/S_0_. To suppress temporal detector noise, the reported value is obtained by averaging multiple frames and taking the spatial mean over a uniform ROI on the flat target surface. Because the indoor background is spatially non-uniform, the ROI is restricted to the target region, and no background pixels are included; the instrumental polarization floor was independently calibrated using an isothermal Lambertian black surface filling the field of view (b = 0.0051) and applied consistently to both materials; the predicted DoLP is formed by the empirical combination DoLP = √(DoLP_phys^2^ + b^2^).

To quantify the repeatability and verify the robustness of the measured polarization signatures, three independent repeated measurements were performed for every material–temperature–angle condition, using the same DoFP camera, target ROI, observation geometry, and calibration procedure. Each measurement was collected after thermal equilibrium, and the Stokes parameters and DoLP were reconstructed from the four polarized orientation images.

### 3.2. Experimental Scheme

#### 3.2.1. Target Selection

**Figure 3:**
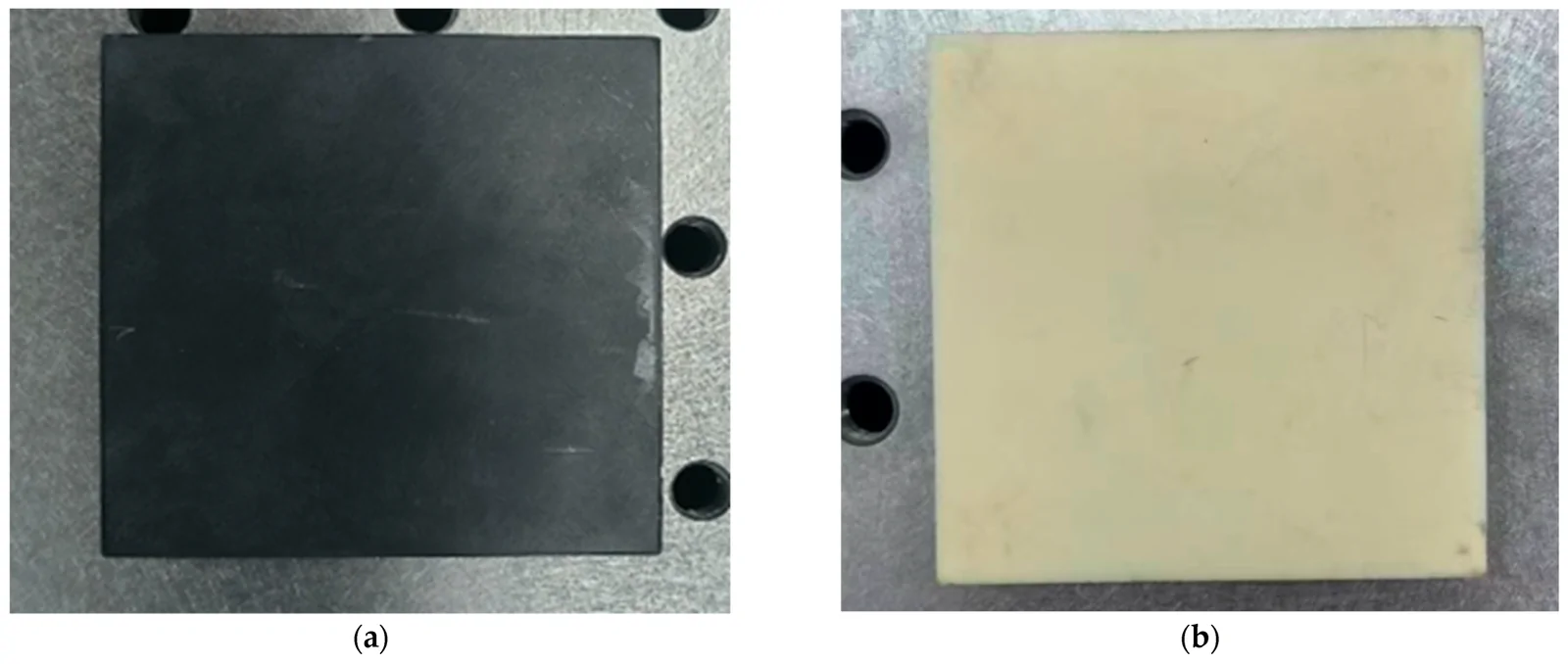
Photographs of the experimental materials: (**a**) silicon carbide; (**b**) alumina.

Two block targets with different microscopic roughness and complex refractive indices were selected (each measuring 50 mm × 50 mm × 10 mm): silicon carbide and alumina (Figure 3). Silicon carbide is a moderately rough, covalently bonded ceramic whose LWIR response is dominated by a strong surface-phonon (Reststrahlen) band. Alumina is a relatively rough, diffuse polycrystalline ceramic that behaves predominantly as a dielectric. The wavelength-dependent optical constants of both materials are taken from the literature and integrated over the 8–14 μm band (Table 2), rather than represented by a single nominal *n* and *k*.

The wavelength-dependent optical constants *n*(λ) and *k*(λ) used in the broadband integration were obtained from the literature. For silicon carbide, they are represented by a single-Lorentz-oscillator model of the Reststrahlen band (standard SiC oscillator parameters, [24]) (ε∞ = 6.52, νTO = 797 cm^−1^, νLO = 970 cm^−1^), with a broadened damping factor (γ = 40 cm^−1^) to approximate the polycrystalline sample. For alumina, the tabulated mid-infrared optical constants of a sputtered alumina film [25] were interpolated over the 8–14 μm band. Because these reference data were acquired on films or single crystals rather than on the exact bulk ceramics used here, they are treated as representative spectral inputs, while the GGX roughness scale parameter and reflection weights are calibrated on the 338 K data. Silicon carbide is relatively temperature-sensitive, so its thermo-optic and extinction-temperature coefficients [22] are set to 4.0 × 10^−5^ and 5.0 × 10^−5^ K^−1^; for polycrystalline alumina, they are approximated as zero over the 338–378 K range.

#### 3.2.2. Experimental Environment

The experiment was conducted in an indoor darkroom, with all active infrared light sources switched off throughout. The polarized energy reaching the detector originated mainly from the target’s own infrared emission and from reflection of the cold indoor environmental background. The environmental background temperature was approximately 288 K, and the target was heated by the temperature-controlled stage to 338 K, 358 K, and 378 K to acquire polarization data under multiple temperature conditions. The indoor environment is represented in the model as a uniform cold background at this measured temperature; any residual spatial non-uniformity that is reflected by the target is not represented and is treated as a modeling limitation (Section 5).

## 4. Results and Discussion

### 4.1. Infrared Polarization Images

#### 4.1.1. Silicon Carbide Ceramics

**Figure 4:**
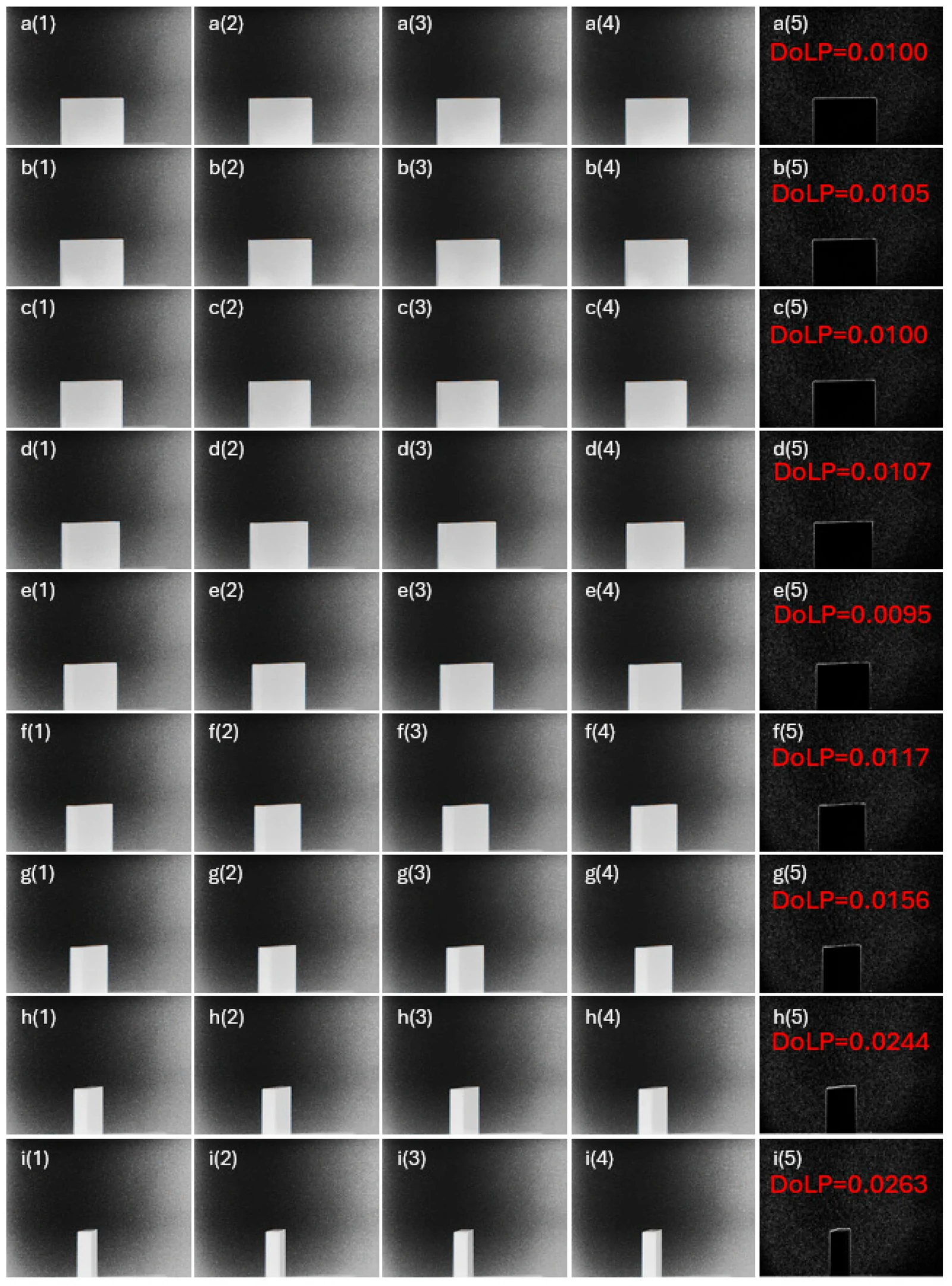
Measured silicon carbide images at 338 K for viewing angles (**a**–**i**) from 0° to 80°: (**1**) 0° polarization image; (**2**) 45° polarization image; (**3**) 90° polarization image; (**4**) 135° polarization image; (**5**) degree-of-polarization image.

**Figure 5:**
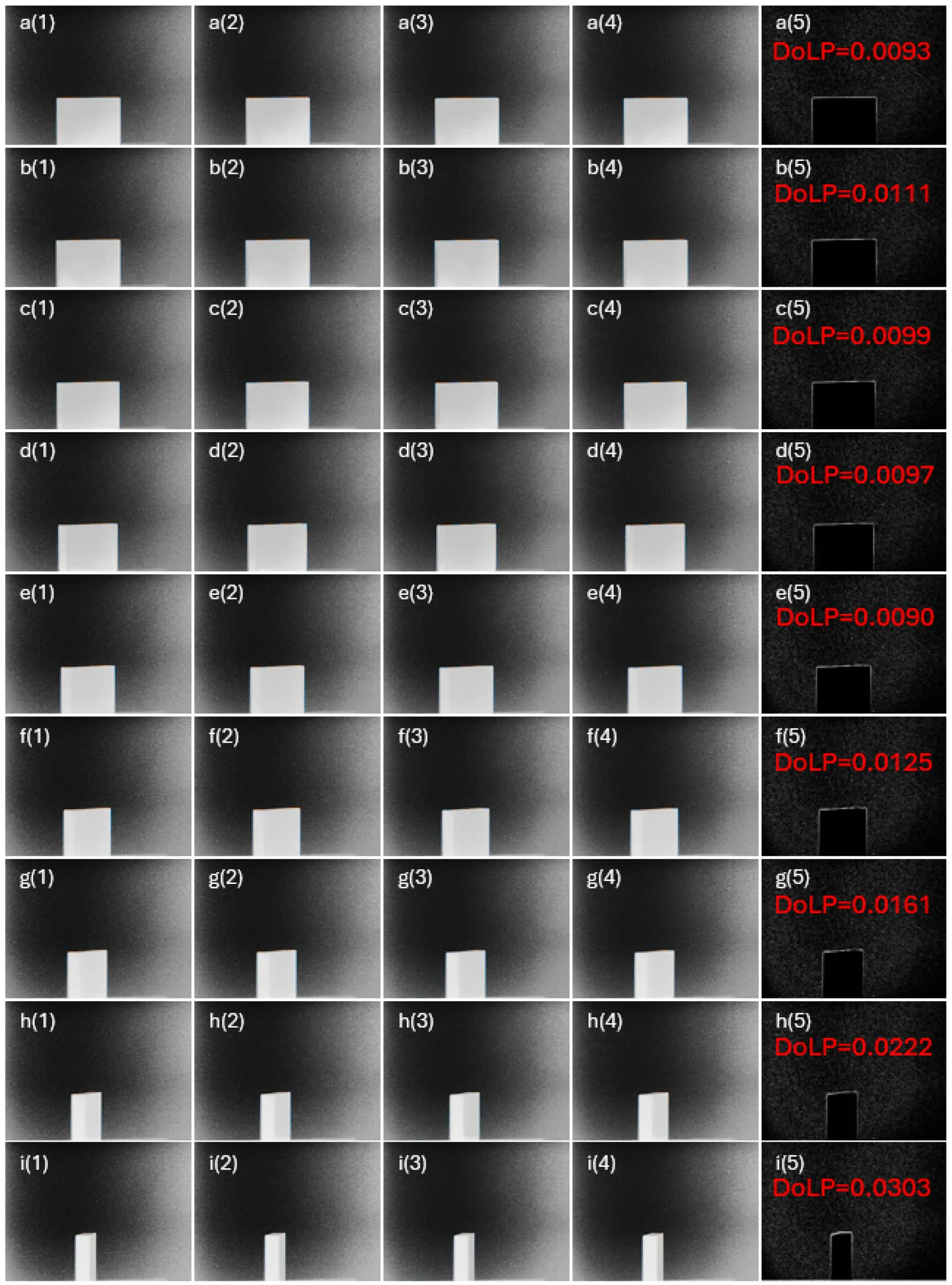
Measured silicon carbide images at 358 K for viewing angles (**a**–**i**) from 0° to 80°: (**1**) 0° polarization image; (**2**) 45° polarization image; (**3**) 90° polarization image; (**4**) 135° polarization image; (**5**) degree-of-polarization image.

**Figure 6:**
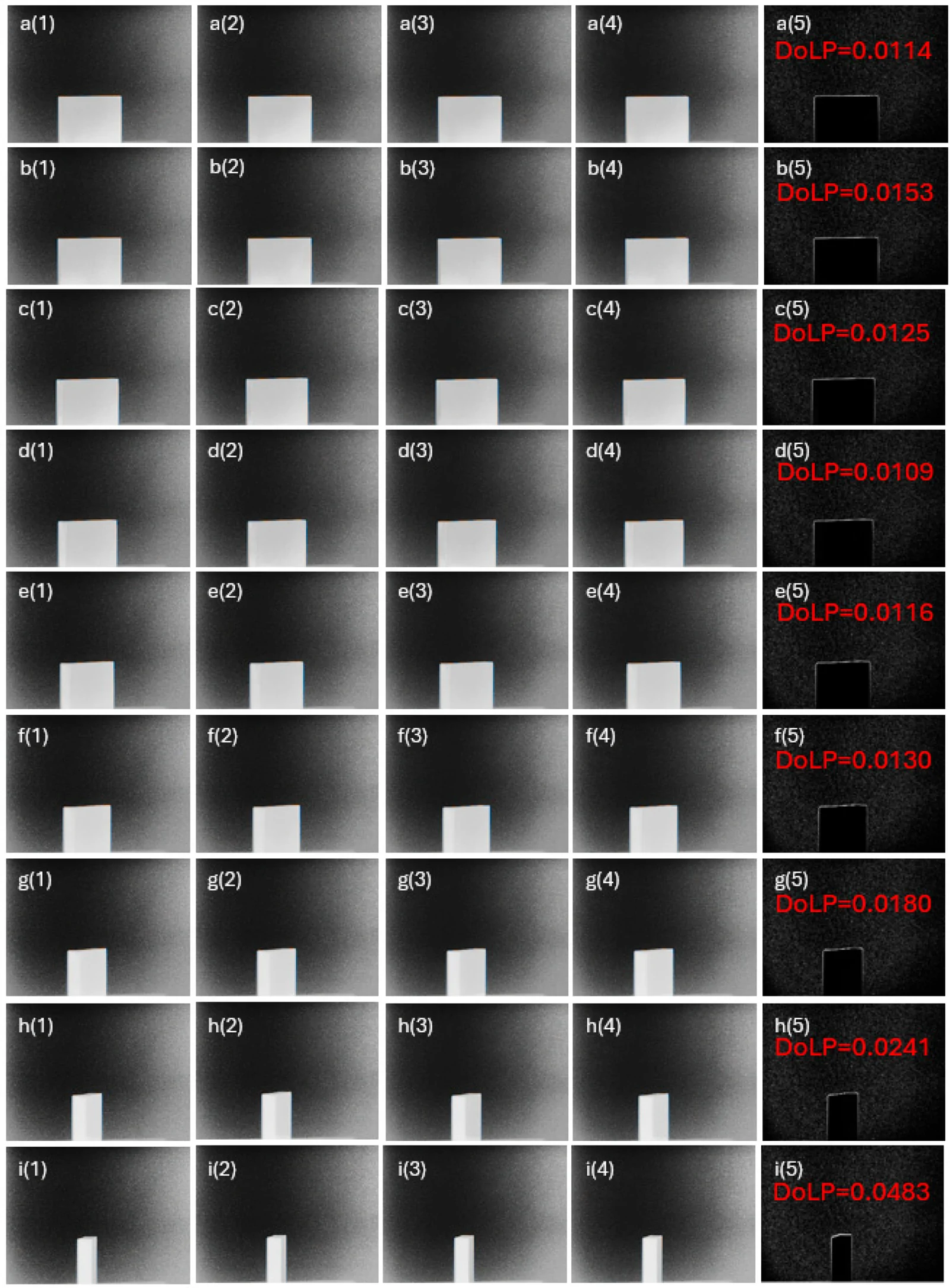
Measured silicon carbide images at 378 K for viewing angles (**a**–**i**) from 0° to 80°: (**1**) 0° polarization image; (**2**) 45° polarization image; (**3**) 90° polarization image; (**4**) 135° polarization image; (**5**) degree-of-polarization image.

Figure 4, Figure 5 and Figure 6 present the infrared polarization images of the silicon carbide ceramic at different temperatures and viewing angles. The temperatures were set to 338 K, 358 K, and 378 K, and the viewing angles to 0°, 10°, 20°, 30°, 40°, 50°, 60°, 70°, and 80°.

As can be seen in Figure 4, the radiating area of the silicon carbide decreases as the viewing angle increases, while the degree of polarization increases with the viewing angle; the increase is modest from 0° to 40° and becomes markedly steeper beyond 50°. At 338 K, the maximum value of 0.0263 is reached at the 80° viewing angle.

As can be seen in Figure 5, the radiating area of the silicon carbide decreases and the degree of polarization increases as the viewing angle increases, following a trend similar to that at 338 K. At 358 K, the maximum value of 0.0303 is reached at the 80° viewing angle.

As can be seen in Figure 6, the radiating area decreases and the degree of polarization increases with the viewing angle, following a trend similar to that at 338 K. At 378 K, the maximum value of 0.0483 is reached at the 80° viewing angle.

Overall, the LWIR degree of polarization of the silicon carbide ceramic increases with viewing angle: the increase is insignificant below 40° and becomes markedly steeper beyond 50°; the degree of polarization also increases with rising temperature.

#### 4.1.2. Alumina Ceramics

**Figure 7:**
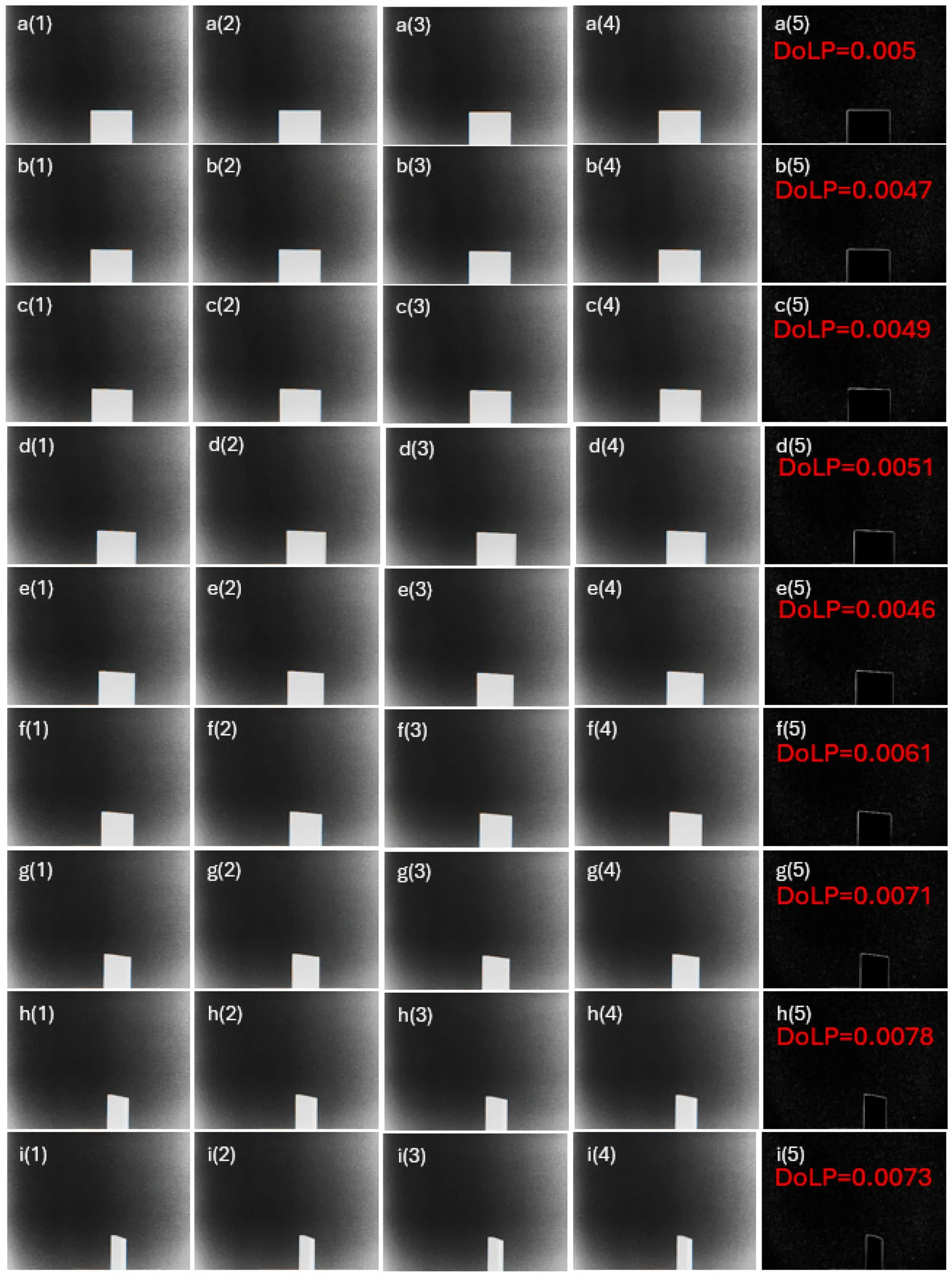
Measured alumina images at 338 K for viewing angles (**a**–**i**) from 0° to 80°: (**1**) 0° polarization image; (**2**) 45° polarization image; (**3**) 90° polarization image; (**4**) 135° polarization image; (**5**) degree-of-polarization image.

**Figure 8:**
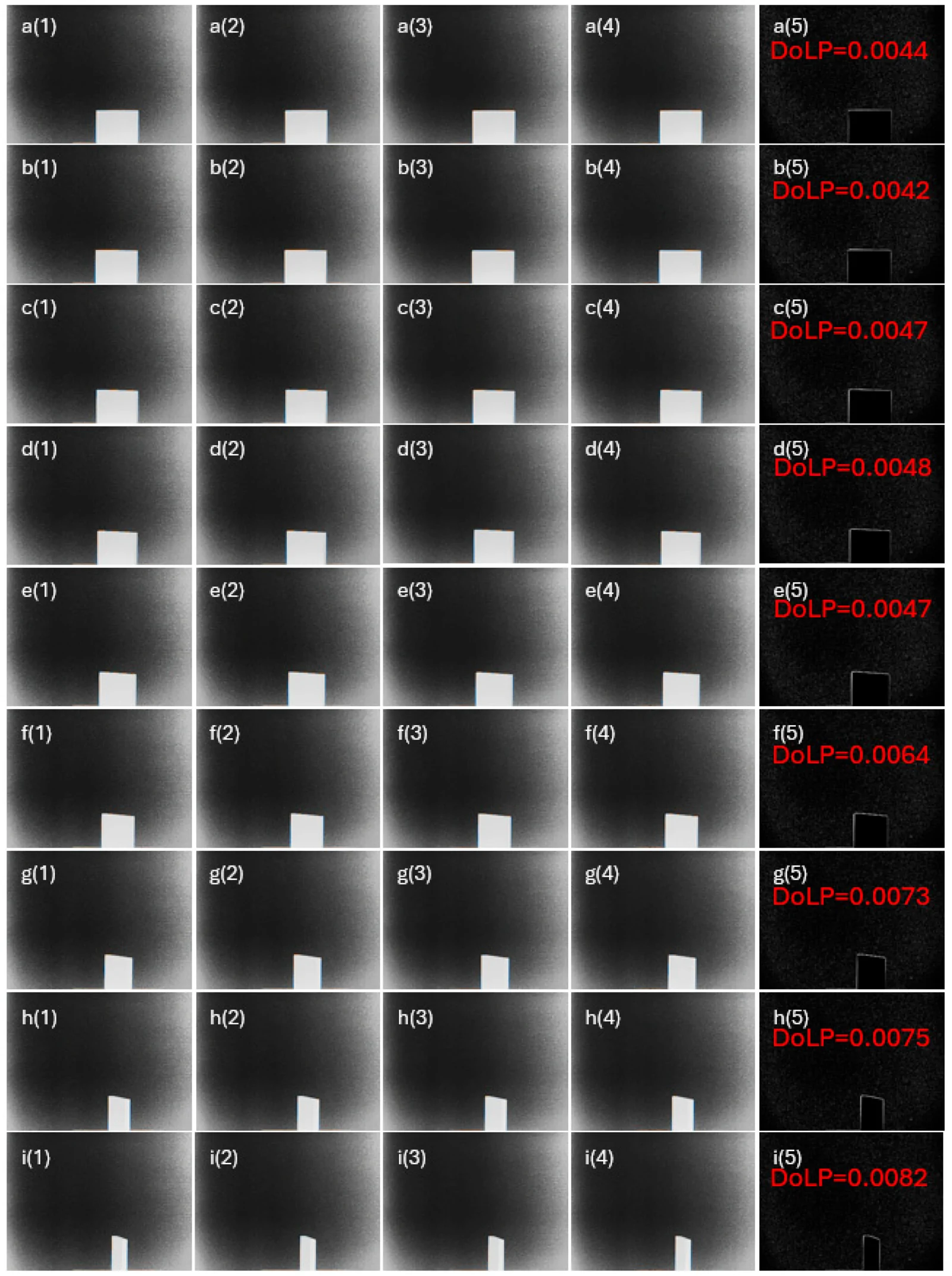
Measured alumina images at 358 K for viewing angles (**a**–**i**) from 0° to 80°: (**1**) 0° polarization image; (**2**) 45° polarization image; (**3**) 90° polarization image; (**4**) 135° polarization image; (**5**) degree-of-polarization image.

**Figure 9:**
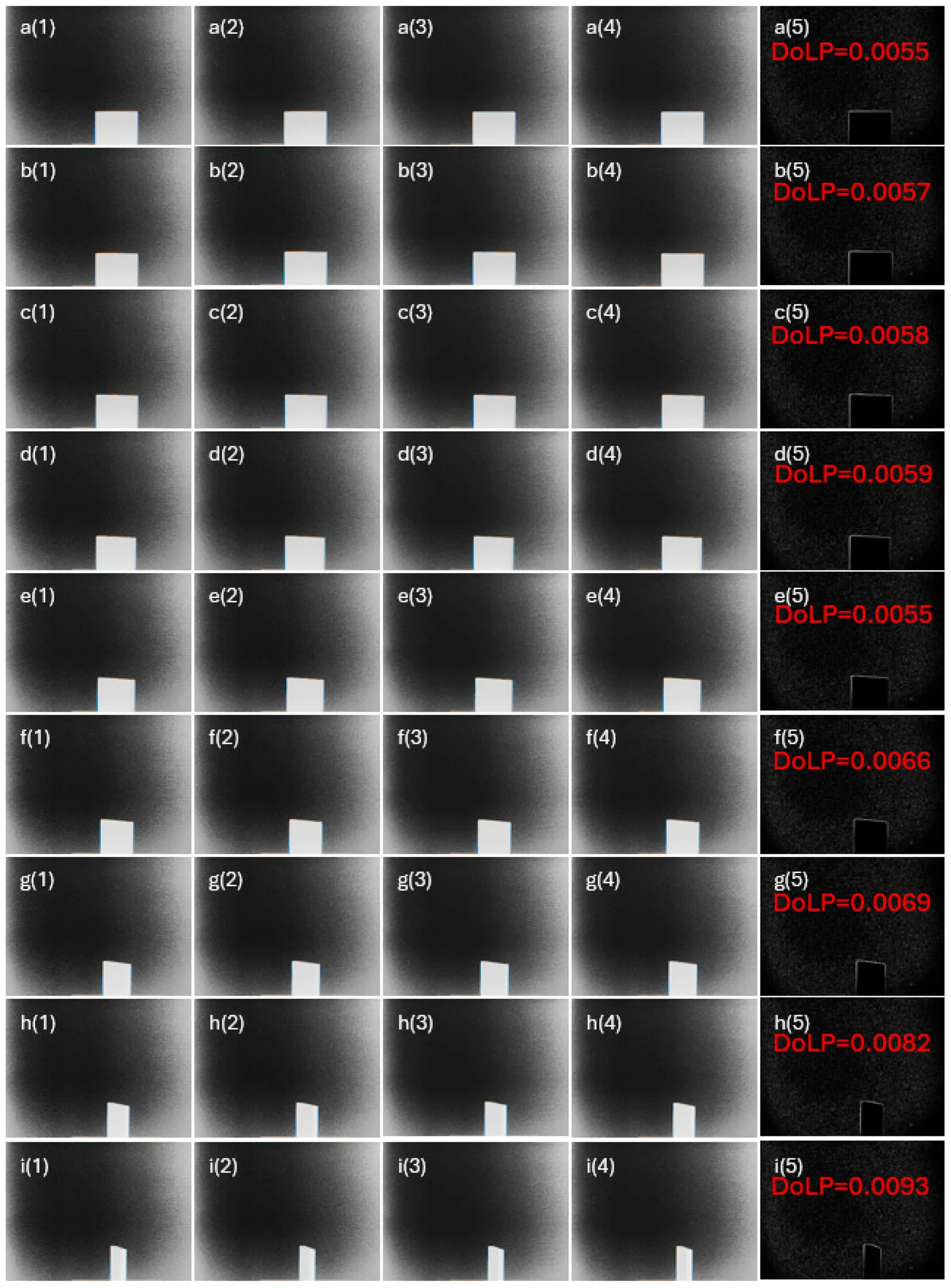
Measured alumina images at 378 K for viewing angles (**a**–**i**) from 0° to 80°: (**1**) 0° polarization image; (**2**) 45° polarization image; (**3**) 90° polarization image; (**4**) 135° polarization image; (**5**) degree-of-polarization image.

Figure 7, Figure 8 and Figure 9 present the infrared polarization images of the alumina ceramic at different temperatures and viewing angles. The temperatures were set to 338 K, 358 K, and 378 K, and the viewing angles to 0°, 10°, 20°, 30°, 40°, 50°, 60°, 70°, and 80°.

As can be seen in Figure 7, the radiating area of the alumina decreases as the viewing angle increases, and the degree of polarization varies with the viewing angle; the increase is modest from 0° to 40° and becomes markedly steeper beyond 50°. At 338 K, the maximum value of 0.0078 is reached at the 70° viewing angle, but the degree of polarization decreases to 0.0073 at 80°.

As can be seen in Figure 8, the radiating area of the alumina decreases as the viewing angle increases, and the degree of polarization increases with the viewing angle; the increase is modest from 0° to 40° and becomes markedly steeper beyond 50°. At 358 K, the maximum value of 0.0082 is reached at the 80° viewing angle.

As can be seen in Figure 9, the radiating area decreases as the viewing angle increases, and the degree of polarization increases with the viewing angle; the increase is modest from 0° to 40° and becomes markedly steeper beyond 50°. At 378 K, the maximum value of 0.0093 is reached at the 80° viewing angle.

The LWIR degree of polarization of the alumina ceramic increases with viewing angle, with an insignificant increase below 40° and a markedly steeper increase beyond 50°; it also increases with rising temperature. Only at 338 K does the maximum measured degree of polarization occur at the 70° viewing angle, whereas under the other two temperature conditions, the maximum occurs at 80°.

The measured data are subsequently used to validate and compare different polarization-degree simulation models.

### 4.2. Polarization-Degree Simulation Comparison and Error Analysis

**Figure 10:**
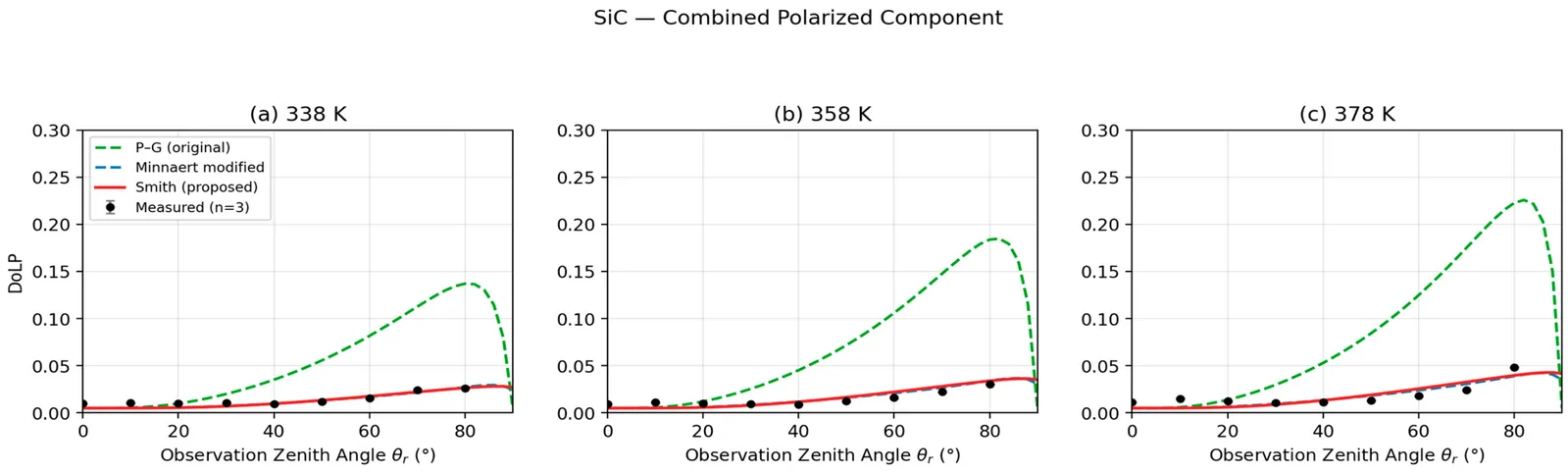
Comparison of measured and simulated DoLP for silicon carbide at (**a**) 338 K, (**b**) 358 K, and (**c**) 378 K. Symbols denote DoFP measurements; error bars show the repeatability standard deviation (*n* = 3); curves are the original P–G (smooth-interface Fresnel emission), Minnaert-modified, and proposed Smith models under the common broadband integration.

**Figure 11:**
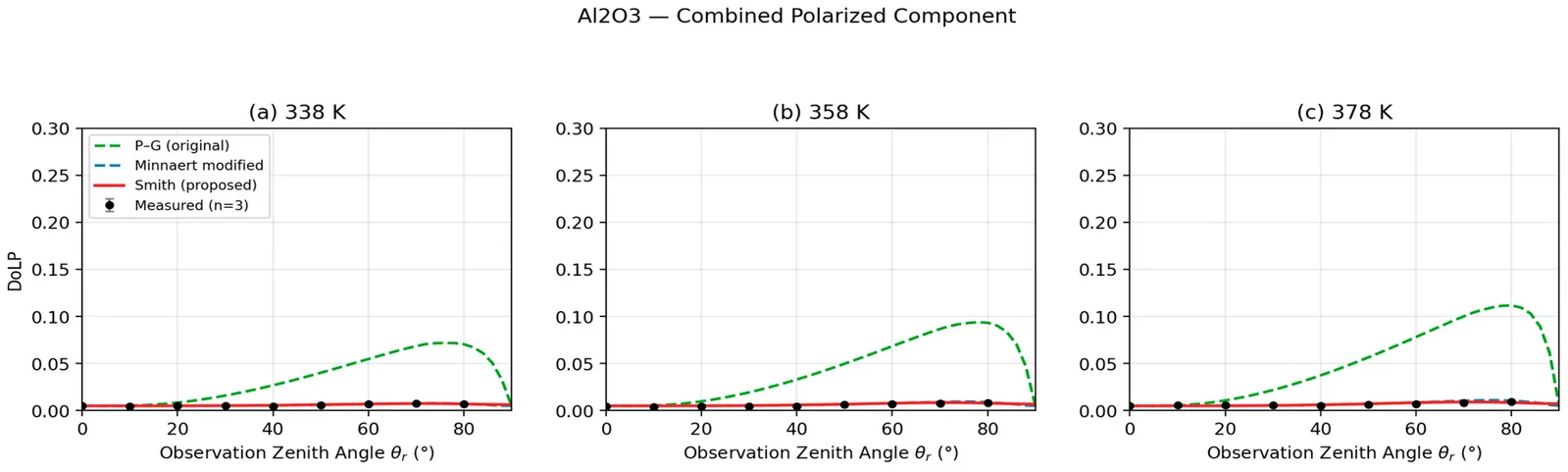
Comparison of measured and simulated DoLP for alumina at (**a**) 338 K, (**b**) 358 K, and (**c**) 378 K. Symbols denote DoFP measurements; error bars show the repeatability standard deviation (*n* = 3); curves are the original P–G, Minnaert-modified, and proposed Smith models under the common broadband integration.

MATLAB R2023a (The MathWorks, Inc., Natick, MA, USA) was used to simulate the P–G model, the Minnaert-modified p-BRDF model, and the Smith-pBRDF model, obtaining the infrared degree of polarization of the different materials at multiple temperatures and viewing angles, and the results were compared with the measured data. The comparison results for silicon carbide and alumina are shown in Figure 10 and Figure 11, respectively.

Three models are compared: the original Priest–Germer (P–G) model, the Minnaert-modified p-BRDF model, and the proposed Smith-pBRDF model. The original P–G model follows its original emission treatment: the directional emissivity is obtained from the smooth-interface Fresnel relation ε = 1 − R(θ) without the microfacet normal distribution or DHR, and it has no fitted parameters; as shown below, this treatment overestimates the DoLP at large angles. The Minnaert model uses a Gaussian NDF with a visibility term and an empirical Minnaert term within the DHR emission module (five parameters). The proposed Smith model uses the GGX NDF with the closed-form Smith masking function within the DHR emission module, with no empirical term (three parameters). All models use the broadband spectral integration (Section 2.2.3) and the same instrument floor (b = 0.0051). The comparison therefore assesses the three models on equal terms, with the original P–G retaining its genuine smooth-interface emission treatment.

To eliminate the subjective deviation that visual comparison of graphs may introduce, the root-mean-square error (RMSE) was adopted to quantitatively evaluate the theoretical fidelity of the three models. Using the previously obtained experimental datasets for different materials and temperatures, the simulation error of each model was calculated. The results are summarized in Table 3; the corresponding mean absolute error, maximum absolute error, and coefficient of determination are reported in Table 4.

For a fair comparison, the Minnaert and Smith models were calibrated on the 338 K data with the same constrained least-squares optimizer (minimizing the sum of squared residuals between the nine-angle predicted and measured DoLP), the bounds ks, kd, kv ∈ [0, 1] and σ ∈ [0.02, 1], and multiple starting points to avoid local minima; the parameters were then held fixed, and the 358 K and 378 K values are retrospective temperature hold-out predictions. Because the original data had been inspected during model development, this is described as a retrospective rather than a strictly blind hold-out, while the three-repeat measurements (Section 4.3) provide an independent assessment of measurement uncertainty. The original P–G model requires no calibration: its emission polarization is fixed by the smooth-interface Fresnel relation. The free-parameter counts are listed in Table 5: the Smith model uses three parameters, the Minnaert model uses five (an empirical Minnaert term adds a weight and an exponent), and the original P–G model uses none.

For silicon carbide, the Minnaert and Smith models both reproduce the angular signature well, with RMSE of 0.0032–0.0033, 0.0041–0.0045 and 0.0065–0.0070 across the three temperatures and positive R^2^ values of 0.57–0.72. By contrast, the original P–G model strongly overestimates the DoLP (RMSE 0.055–0.089, strongly negative R^2^), because its smooth-interface Fresnel emission neglects the depolarization caused by microfacet slope dispersion and diverges at large angles. The Smith model reaches this accuracy with three parameters and no empirical term, and attains the lowest AICc (−59.33) and BIC (−68.54) among the three; the original P–G model gives the highest AICc (−24.11), and the five-parameter Minnaert model is intermediate (−23.60). The closed-form Smith masking therefore provides the most favorable accuracy–complexity trade-off for this surface-reflection-dominated material.

The empirical Minnaert term has the form f_v = (c + 1)/(2π)(cos θ_i cos θ_r)^c, weighted by kv; for the classical Minnaert law, the constant k = c + 1 lies in [0, 1] (c ∈ [−1, 0]). The Minnaert model improves the agreement through this term, giving a low RMSE on silicon carbide; however, the fitted exponent c = 5.56 lies well outside the classical Minnaert interval, and the additional weight and exponent must be re-determined for each material. This indicates that the empirical term is a flexible but material-specific correction rather than a general physical mechanism, whereas the closed-form Smith masking follows directly from the surface statistics, keeps the parameters within their physical bounds, and yields a lower information criterion.

For alumina, the Minnaert and Smith models again reproduce the measurements well (RMSE 0.0004–0.0011), whereas the original P–G model overestimates the DoLP (RMSE 0.036–0.056), confirming the inadequacy of the smooth-interface Fresnel treatment for this diffuse ceramic. The Smith model is the most accurate at 358 K and 378 K (RMSE 0.0008) and attains the lowest AICc (−95.89) and BIC (−105.10); the Minnaert model is slightly weaker at 378 K (R^2^ 0.18). Because the measured DoLP is close to the instrument floor and the surface is near-Lambertian, the absolute DoLP is small; the Smith model nevertheless reproduces these weak values with three parameters and the lowest information criterion, while the original P–G model cannot.

**Figure 12:**
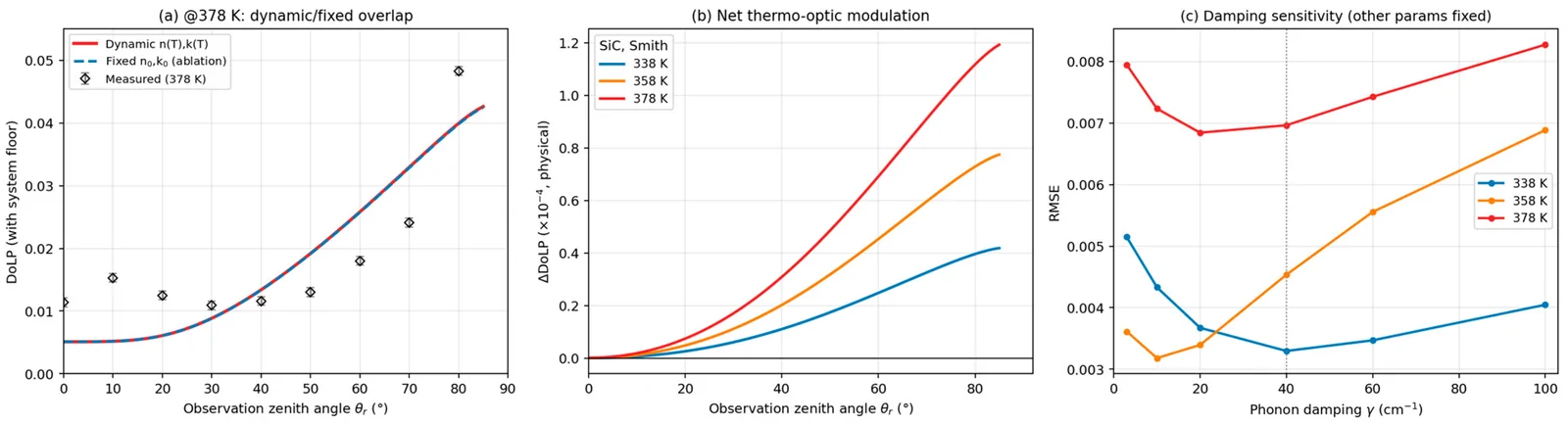
Fixed-versus-dynamic thermo-optic ablation and sensitivity analysis for silicon carbide (Smith model): (**a**) the dynamic and fixed curves at 378 K nearly coincide at the displayed scale; (**b**) the net thermo-optic modulation ΔDoLP grows with ΔT and grazing angle but remains at the 10^−5^–10^−4^ level; (**c**) the Smith RMSE stays within about 0.003–0.008 as the phonon damping is varied over 3–100 cm^−1^ (other parameters fixed), far below the original P–G model.

To isolate the contribution of the thermo-optic term and to verify that the result is not driven by the optical-constant assumptions, Figure 12 reports a fixed-versus-dynamic ablation and a sensitivity analysis. The dynamic and fixed curves nearly coincide at the displayed scale at 378 K (Figure 12a), and the net thermo-optic modulation is at most about 1.2 × 10^−4^ (Figure 12b); the Smith RMSE stays within about 0.003–0.008 as the phonon damping is varied over 3–100 cm^−1^ (Figure 12c), still far below the original P–G model.

### 4.3. Repeatability and Reproducibility Assessment

**Figure 13:**
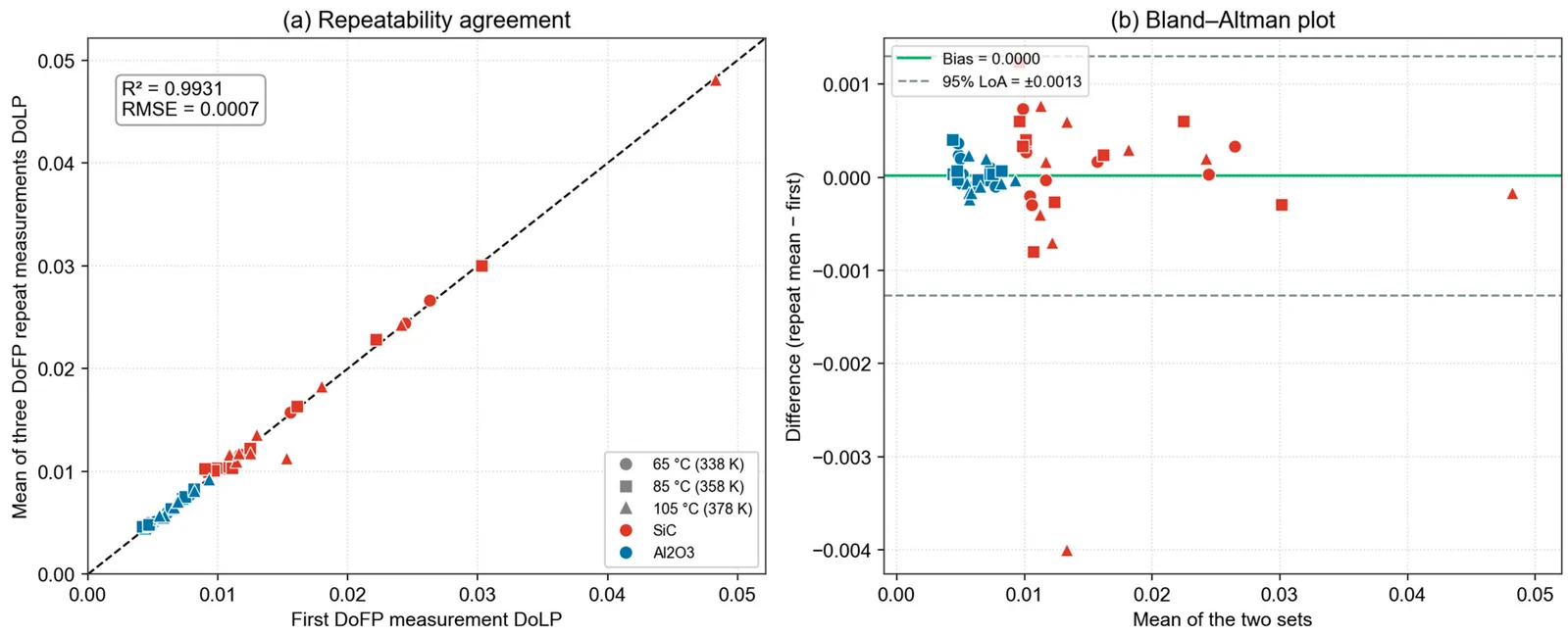
Repeatability agreement of the DoFP measurements: (**a**) point-by-point comparison of the first measurement and the mean of three repeat measurements with the identity line; (**b**) Bland–Altman plot of differences against the mean, showing the bias and the 95% limits of agreement.

The repeated measurements were used to assess the measurement uncertainty (Figure 13). Across the 54 material–temperature–angle points, the within-method repeatability (standard deviation of the three replicates) was 1.9 × 10^−4^ on average (maximum 7.1 × 10^−4^). The agreement between the first measurement and the mean of the three repeat measurements was high: the pooled bias was 2 × 10^−5^ (essentially zero), with an RMSE of 6.5 × 10^−4^, MAE of 3.3 × 10^−4^, and coefficient of determination R^2^ = 0.993. The Bland–Altman 95% limits of agreement were ±1.3 × 10^−3^; one of the 54 points (1.9%) fell outside these limits. This point corresponds to the 10° observation of SiC at 378 K, where the first measurement contained a local positive excursion, while the repeat mean (0.0113) remained consistent with the neighboring angles. Overall, the close agreement between the first and repeat measurements supports the repeatability of the reported angular and temperature-dependent polarization signatures and provides an empirical uncertainty bound for the weak DoLP values.

## 5. Conclusions and Outlook

To address the problems of conventional long-wave infrared polarization p-BRDF models—namely their tendency toward energy divergence at large observation angles, their reliance on empirical parameters, and their neglect of the thermo-optic effect—this paper proposes a polarized reflection model based on the Smith masking function (the Smith-pBRDF model) and conducts experimental comparison and validation of the model by building an infrared polarization goniometric experimental platform.

In this study, the Smith masking function is used to correct the divergence of the P–G model at large angles, and the reliance on fitting parameters is reduced under the constraint of energy conservation. The rise of DoLP with temperature is governed primarily by the Planckian target–background radiance contrast; the thermo-optic and extinction-temperature coefficients provide a secondary, high-order refinement of the complex refractive index, retained because it explicitly represents the assumed temperature dependence of the optical constants. In addition, the model computes the emission polarization components through two-dimensional DHR hemispherical integration in order to describe the macroscopic depolarization of rough surfaces induced by microfacet slope dispersion and to remedy the overestimation of the emission degree of linear polarization under the conventional smooth approximation used by the original P–G model. Experimental comparison across materials and temperatures shows that the original P–G model, with its smooth-interface Fresnel emission, overestimates the DoLP by roughly an order of magnitude; the five-parameter Minnaert model corrects this trend but relies on an empirical term, whereas the proposed Smith model attains the lowest AICc and BIC for both materials with only three parameters. The model therefore reflects the evolution of target infrared polarization across materials and temperatures with limited reliance on empirical parameters, providing a reference for subsequent infrared polarization detection and simulation research.

Limited by experimental conditions and the number of samples, the performance of the proposed model in more complex scenarios remains to be further investigated. In addition, independent surface profilometry was not available, so the roughness parameter is calibrated rather than measured, and a formal parameter confidence interval or parametric prediction interval is not reported; these are acknowledged as limitations, and the measurement error bars, cross-check and sensitivity analyses provide empirical uncertainty bounds instead. Future work will focus mainly on two aspects: first, examining the computational performance of the model on targets with complex morphologies, such as irregular curved surfaces and multi-scale rough surfaces, and second, progressively introducing real measurement factors such as environmental stray-light interference and dynamic background evolution into the simulation modeling, so as to improve the applicability of the model under complex backgrounds. In the future, the theoretical model can be combined with end-to-end simulation of infrared polarization imaging systems to provide an auxiliary analysis tool for the engineering design of infrared polarization detection systems.

## Figures and Tables

**Table 1:** Specifications of the infrared polarization detector.

Parameter	Specification
Detector	Uncooled vanadium-oxide focal-plane-array detector
Resolution	640×512
Pixel size	17 μm×17 μm
Operating waveband	8~14 μm
Polarization orientations	0°,45°,90°,135°
Operating temperature	−40 °C~+60 °C
Frame rate	50 Hz
NETD	$\leq 50\;mK\left(@F/1.0,25\;{}^{\circ}C\right)$

**Table 2:** Parameters of the three models for each material. The original P–G model uses its smooth-interface Fresnel emission treatment and has no fitted parameters (denoted by a dash); the Minnaert model uses five parameters (ks, kd, kv, c, σ), and the Smith model uses three (ks, kd, σ). The Minnaert and Smith parameters are calibrated on the 338 K, nine-angle subset and then held fixed for the 358 K and 378 K predictions. For the Smith model, σ is a dimensionless GGX roughness-scale parameter, whereas for the Minnaert model, it is a Gaussian (Beckmann) roughness parameter. The spectral optical-constant sources and the fixed thermo-optic coefficients are described in Section 3.2.1.

Material	Model	k_s	k_d	k_v	c	Roughness Param.
SiC	P–G (original, smooth)	–	–	–	–	– (no fitted params)
SiC	Minnaert modified	0.327	0.000	0.673	5.561	0.213
SiC	Smith (proposed)	0.950	0.050	–	–	0.528
Al2O3	P–G (original, smooth)	–	–	–	–	– (no fitted params)
Al2O3	Minnaert modified	0.078	0.000	0.922	−0.615	0.067
Al2O3	Smith (proposed)	0.122	0.000	–	–	0.136

**Table 3:** Root-mean-square errors (RMSEs) of the three models. The Minnaert and Smith models are calibrated on the 338 K subset (marked *) with the same optimizer and parameterization; the 358 K and 378 K values are retrospective temperature hold-out predictions after the parameters were frozen. The original P–G model uses the smooth-interface Fresnel emission treatment with no fitted parameters. All models use the same broadband integration and the same instrument floor (b = 0.0051).

Material (Temperature)	P–G Model	Minnaert-Modified p-BRDF	Smith-pBRDF Model
Alumina (338 K) *	0.0362	0.0004	0.0004
Alumina (358 K)	0.0474	0.0009	0.0008
Alumina (378 K)	0.0556	0.0011	0.0008
Silicon carbide (338 K) *	0.0550	0.0032	0.0033
Silicon carbide (358 K)	0.0764	0.0041	0.0045
Silicon carbide (378 K)	0.0894	0.0065	0.0070

**Table 4:** Mean absolute error (MAE), maximum absolute error (Max), and coefficient of determination (R^2^) of the three models; each cell lists MAE/Max/R^2^. The model complexity is compared in Table 5. The 338 K subset used for calibration is marked with *.

Material (Temperature)	P–G Model	Minnaert-Modified p-BRDF	Smith-pBRDF Model
Alumina (338 K) *	MAE 0.0271 Max 0.0632 R^2^ −932.79	MAE 0.0002 Max 0.0010 R^2^ 0.90	MAE 0.0003 Max 0.0011 R^2^ 0.87
Alumina (358 K)	MAE 0.0355 Max 0.0852 R^2^ −1051.53	MAE 0.0008 Max 0.0017 R^2^ 0.64	MAE 0.0007 Max 0.0013 R^2^ 0.73
Alumina (378 K)	MAE 0.0410 Max 0.1025 R^2^ −1946.48	MAE 0.0009 Max 0.0023 R^2^ 0.18	MAE 0.0007 Max 0.0016 R^2^ 0.58
Silicon carbide (338 K) *	MAE 0.0394 Max 0.1108 R^2^ −78.54	MAE 0.0028 Max 0.0053 R^2^ 0.72	MAE 0.0028 Max 0.0054 R^2^ 0.71
Silicon carbide (358 K)	MAE 0.0547 Max 0.1536 R^2^ −121.61	MAE 0.0039 Max 0.0059 R^2^ 0.65	MAE 0.0043 Max 0.0061 R^2^ 0.57
Silicon carbide (378 K)	MAE 0.0646 Max 0.1744 R^2^ −61.51	MAE 0.0059 Max 0.0101 R^2^ 0.67	MAE 0.0064 Max 0.0101 R^2^ 0.62

**Table 5:** Number of free parameters and information criteria (AICc, BIC) evaluated on the 338 K data; the residual variance estimated from the same data is included in the statistical parameter count, and lower values indicate a better accuracy–complexity trade-off. The original P–G model has no fitted parameters but, because its smooth-interface emission overestimates the DoLP, gives the highest information criteria. The Smith model uses three parameters and attains the lowest AICc and BIC for both materials; the five-parameter Minnaert model is intermediate.

Material/Model	Free Params	AICc	BIC
Al_2_O_3_, P–G	0	−31.64	−32.02
Al_2_O_3_, Minnaert	5	−62.76	−103.58
Al_2_O_3_, Smith	3	−95.89	−105.10
SiC, P–G	0	−24.11	−24.48
SiC, Minnaert	5	−23.60	−64.42
SiC, Smith	3	−59.33	−68.54

## Data Availability

The data presented in this study are available from the corresponding author upon reasonable request.

## Abbreviations

The following abbreviations are used in this manuscript:

Al_2_O_3_	Alumina (aluminum oxide)
BRDF	Bidirectional reflectance distribution function
DoFP	Division-of-focal-plane
DoLP	Degree of linear polarization
DHR	Directional–hemispherical reflectance
LWIR	Long-wave infrared
pBRDF	Polarized bidirectional reflectance distribution function
P-G	Priest–Germer
RMSE	Root-mean-square error
RMS	Root mean square
SiC	Silicon carbide

## References

[1] Wang L. (2022). Analysis of Polarization Characteristics of Target Surfaces Based on the pBRDF Model. Master’s Thesis.

[2] Yuan S., Ouyang M., Fu Y., Yang C. (2024). Bionic polarization vision-based solid/gas heat-source recognition technology using deep learning. Infrared Laser Eng..

[3] Wang Y., Liu H., Shi Z., Tong Q. (2025). Research progress on infrared polarization imaging detection technology. J. Shaanxi Norm. Univ. (Nat. Sci. Ed.).

[4] Shi D., Liu L., Huang F., Huang F., Wang X.Z. (2024). Research progress in infrared polarization imaging technology. Laser Optoelectron. Prog..

[5] Yang M., Sun X., Chen D., Xiu P., Yang J., Mao H. (2024). An improved infrared polarization model considering the volume scattering effect for coating materials. Infrared Phys. Technol..

[6] Gong L., Yu J., Yang Z., Li Y., Yang L., Yu Y., Wu Z., Wang L. (2024). Infrared polarization model optimization and radiation characteristics of camouflage coatings. Infrared Phys. Technol..

[7] Liu Y., Shi H., Jiang H., Li Y., Wang C., Liu Z., Li G. (2020). Investigation on infrared polarization characteristics of rough target surfaces. Chin. Opt..

[8] Torrance K.E., Sparrow E.M., Birkebak R.C. (1966). Polarization, directional distribution, and off-specular peak phenomena in light reflected from roughened surfaces. J. Opt. Soc. Am..

[9] Flynn D.S., Alexander C. (1995). Polarized surface scattering expressed in terms of a bidirectional reflectance distribution function matrix. Opt. Eng..

[10] Priest R.G., Meier S.R. (2002). Polarimetric microfacet scattering theory with applications to absorptive and reflective surfaces. Opt. Eng..

[11] Chen W., Wang S., Jin W., Li J. (2014). Research on infrared polarization characteristics based on the polarized microfacet theory. J. Infrared Millim. Waves.

[12] Hyde M.W., Schmidt J.D., Havrilla M.J. (2009). A geometrical optics polarimetric bidirectional reflectance distribution function for dielectric and metallic surfaces. Opt. Express.

[13] Liu H., Zhu J., Wang K. (2015). Correction of the geometric attenuation factor of the bidirectional reflectance distribution function based on the random-surface microfacet theory. Acta Phys. Sin..

[14] Zhu D., Hu Z., Wang F., Jin X., He F. (2022). A spontaneous-emission polarization model of metal surfaces corrected by a masking function. Laser Optoelectron. Prog..

[15] Wang F., Yi L., Wang F., Zhu D., Lei J., Luan Q., Liu Y. (2021). Polarized bidirectional reflectance distribution function of metal surfaces optimized by diffuse reflection. Acta Opt. Sin..

[16] Wang W. (2025). Modeling and Experimental Analysis of Target Infrared Polarization Characteristics Under a Sky Background. Master’s Thesis.

[17] Yang Z., Lu G., Zhang Z., Song J. (2021). Analysis of target infrared polarization characteristics in a thermal radiation environment. Acta Opt. Sin..

[18] Yan K., Liu H., Shi Z., Pang M., Cui Y., Tong Q. (2025). An infrared DoLP calculation model with parameter instantiation for high-temperature rough surfaces. Infrared Phys. Technol..

[19] Ming K., Zong F., Li K., Wang X., Xie G., Gao G., Huang S., Zhan J., Li G., Jiang H. (2025). LWIR polarization characteristics of targets in sea fog via an enhanced p-BRDF model. Opt. Express.

[20] Smith B. (1967). Geometrical shadowing of a random rough surface. IEEE Trans. Antennas Propag..

[21] Walter B., Marschner S.R., Li H., Torrance K.E. Microfacet models for refraction through rough surfaces. Proceedings of the 18th Eurographics Conference on Rendering Techniques (EGSR’07).

[22] Ghosh G. (1998). Handbook of Thermo-Optic Coefficients of Optical Materials with Applications.

[23] Howell J.R., Mengüc M.P., Daun K.J. (2020). Thermal Radiation Heat Transfer.

[24] NSM Archive Physical Properties of Semiconductors: Silicon Carbide (SiC).

[25] Kischkat J., Peters S., Gruska B., Semtsiv M., Chashnikova M., Klinkmüller M., Fedosenko O., Machulik S., Aleksandrova A., Monastyrskyi G. (2012). Mid-infrared optical properties of thin films of aluminum oxide, titanium dioxide, silicon dioxide, aluminum nitride, and silicon nitride. Appl. Opt..

